# A Functional Variant of *NEDD4L* Is Associated with Obesity and Related Phenotypes in a Han Population of Southern China

**DOI:** 10.3390/ijms14047433

**Published:** 2013-04-02

**Authors:** Yu-Lin Wang, Hui-Ying Liang, Yun-He Gao, Xue-Ji Wu, Xi Chen, Bing-Ying Pan, Xue-Xi Yang, Hua-Zhang Liu

**Affiliations:** 1 Guangzhou Center for Disease Control and Prevention, Guangzhou 510440, China; E-Mails: wangyL1858@sina.com (Y.-L.W.); lianghuiying@hotmail.com (H.-Y.L.); janeyee@126.com (X.-J.W.); jessicaxx@126.com (X.C.); jgk@gzcdc.org.cn (B.-Y.P.); 2 Guangzhou Women and Children Medical Center, Guangzhou 510623, China; E-Mail: gaoliangmidi@yahoo.cn; 3 School of Biotechnology, Southern Medical University, Guangzhou 510515, China

**Keywords:** *NEDD4L*, genetic diversity, obesity

## Abstract

*NEDD4L* is a candidate gene for hypertension, both functionally and genetically. Recently, studies showed evidence for the association of *NEDD4L* with obesity, a key intermediate phenotype in hypertension. To further investigate the relationship between *NEDD4L* and body mass-related phenotypes, we genotyped three common variants (rs2288774, rs3865418 and rs4149601) in a population-based study of 892 unrelated Han Cantonese using the Sequenom MALDI-TOF-MS platform. Allele frequencies and genotype distribution were calculated in lean controls and overweight/obese cases and analyzed for association by the Chi-squared test and Logistic regression. Linear regression analysis was used to analyze the effect of individual genotypes on quantitative traits. Multivariate analyses demonstrated that the minor allele of rs4149601(A = 20.9%) was associated with a 2.60 kg, 2.78 cm and 0.97 kg/m^2^ decrease per allele copy in weight, waist and BMI, respectively. Carriers of this allele also had a significant lower risk of overweight/obesity (*p* < 0.0001, OR = 0.52, 95% CI: 0.37–0.74) as compared to non-carriers. However, no significant association between genotypes at rs2288774 and rs3865418 and covariate-adjusted overweight/obesity or any related phenotypes was observed. These results suggested that the functional variant of *NEDD4L*, rs4149601, may be associated with obesity and related phenotypes, and further genetic and functional studies are required to understand its role in the manifestation of obesity.

## 1. Introduction

Numerous linkage studies have indicated *NEDD4L* as a candidate gene for essential hypertension among different populations [1–4]. Although the precise pathways and biological mechanisms underlying these associations have yet to be established, according to recent research [5,6], two potential separate mechanisms are worthy of consideration: first, acting directly via the epithelial Na^+^ channel (ENaC)-*NEDD4L*-proteasome system [7], *NEDD4L* is the key link of this system [8], which plays an important role in the regulation of blood pressure (BP) [9]; second, acting indirectly via key intermediate phenotypes of BP, such as obesity [10] and salt sensitivity [6], both of which are major risk factors for the development of hypertension [11,12]. Given the complex etiology and pathophysiology of hypertension, dissecting the intermediate phenotype of BP and unraveling intermediate mechanisms has been suggested to be more comprehensible [13,14].

Recently, a common polymorphism located at exon 1 (rs4149601) of the *NEDD4L* gene was shown to be associated with obesity in Kazakh [10] and to not be associated in the same group for another common polymorphism located at intron 12 (rs3865418) [15]. No other studies and replications are available on the issue of whether *NEDD4L* genetic variation is a contributing factor to the risk of obesity. These common variants may also become involved in obesity-related phenotypes. Particularly, BMI is a highly heritable phenotype, but robust associations of genetic polymorphisms to BMI or other obesity-related phenotypes have been difficult to establish. However, the effect of the *NEDD4L* genetic variation on obesity-related phenotypes remains unclear, and there was no association analysis of common variants in *NEDD4L* with BMI in non-Asian populations.

Our study, therefore, focused on the association of genetic variation in *NEDD4L* with obesity and 11 related phenotypes in a genetically isolated Han Chinese population, which we have previously used successfully to identify single-nucleotide polymorphisms (SNPs) associated with obesity-related phenotypes. In this study, three common variants (rs4149601, rs3865418 and rs2288774) were selected from dbSNP and from NCBI, based on their position in the gene, minor allele frequency and previous studies.

## 2. Results and Discussion

A test for Hardy-Weinberg Equilibrium (HWE) suggested that the genotypes for all the SNPs were in Hardy-Weinberg proportions, and there was no deviation from HWE among normal weight subjects (*p* = 0.79, 0.71 and 0.49 for rs2288774, rs3865418 and rs4149601, respectively). The frequencies of minor alleles in the whole study group were 20.9% for rs4149601, 35.3% for rs3865418 and 38.9% for rs2288774, respectively. It is in good agreement with the Han Chinese in Beijing (20.0%, 35.6% and 32.2%, respectively). However, blacks and Caucasians have quite different frequencies (Table 1). Linkage disequilibrium (LD) analysis using Haploview revealed that the three SNPs spanned three different LD blocks (*D*’ = 0.09–0.72, *r*^2^ = 0.001–0.44). As the SNPs can’t substitute each other, we decided to describe in “Results” the findings for all three SNPs.

### 2.1. Association Analysis of Genetic Variants in *NEDD4L* with Overweight/Obesity

Allele frequencies at rs4149601 showed significant difference between healthy lean controls and overweight/obese cases, with the *p*-value being 0.0002. The difference remained statically significant even after correction for multiple testing by Bonferroni correction (×3, *p*-value 0.0006) (Table 2). Correspondingly, rs4149601 was significantly associated with overweight/obesity (*p* < 0.0001, OR = 0.51, 95% CI: 0.37–0.71). Significant values were also obtained in the additive model analysis after adjustment for age, sex, smoking, hypertensive status, alcohol consumption and exercise habit (*p* < 0.0001, OR = 0.52, 95% CI: 0.37–0.74). None of the other two selected SNPs showed any significant association with overweight/obesity. Results of association analysis are summarized in Table 3.

### 2.2. Analysis of Obesity Related Phenotypes and Genotypes at Genetic Variants in *NEDD4L*

Tables 4, S1, and S2 present the relationship of three selected SNPs with 11 obesity-related phenotypes. One of the three SNPs, rs4149601, had a significant association with weight, waist and BMI (*p* = 0.0003, 0.0015 and 0.0012, respectively) with an allele-dose effect. Carriers of the minor allele had, on average, 2.60 kg decreased weight, 2.78 cm decreased waist and 0.97 kg/m^2^ decreased BMI per allele copy, respectively. For the other explored obesity-related quantitative traits, such as BP, log serum triglycerides (TG), total cholesterol (TC), low-density lipoprotein cholesterol (LDL-c), high-density lipoprotein cholesterol (HDL-c) and glucose levels and the anthropometric variables of height, we observed no evidence for an association with the rs4149601 genotype (Table 4). There were no associations between any of the obesity-related phenotypes and the rs2288774 polymorphism (Table S1) and the rs3865418 polymorphism (Table S2).

*NEDD4L* is a member of the homologous to the E6AP carboxyl terminus (HECT) class of E3 ubiquitin ligases, which plays an important role in the development of lipid disorders [16], central obesity [17] and hypertension [18]. *NEDD4L* is, thus, an attractive candidate gene for susceptibility to hypertension and intermediate blood pressure phenotypes (e.g., obesity [10] and salt sensitivity [6]). The present study is the first population-based study reporting an association between *NEDD4L* genetic variation and obesity or related phenotypes in an independent Han Chinese population. In this study, we first used a case-control approach for analyzing three common SNPs in *NEDD4L* for association with overweight/obesity. Furthermore, we also investigated 11 obesity-related quantitative traits for their association with selected SNPs. We provide evidence that genetic variation of *NEDD4L* may be implicated in the prevalence of overweight/obesity and related phenotypes in Chinese Hans.

Recently published genetic studies in the Kazakh general population corroborate our findings that no significant associations were found between the rs2288774 and rs3865418 polymorphisms and any of the obesity-related phenotypes as quantitative traits [1]; nor was there any association between rs3865418 genotype and obesity when considered as an affection status [15]. However, exploratory evidence for an association of rs4149601 with the decreased risk of being overweight/obese was detected in both the Kazakh and Han Chinese population. As revealed by the odds ratio, carriers of the minor allele (A) of rs4149601 tend to be associated with a decreased susceptibility for being overweight or obese ([OR = 0.52, 95% CI: 0.37–0.74] in our subjects and [OR = 0.67, 95% CI: 0.50–0.91] in the Kazakh population [10]) when compared with subjects who do not carry this allele.

Because no functional studies are available to understand the underlying biological mechanisms of the observed genetic associations, we cannot conclude from our data whether the decreased risk of overweight/obesity that we observed in carriers of the minor allele of rs4149601 is associated with increased or decreased *NEDD4L* activity. However, based on the known functions of the *NEDD4L* gene, one can speculate that the effects of E3-ubiquitin ligase activity on lean mass, as well as fat mass may explain more or less the relation with obesity that we observed [19]. In line with these findings, we also observed that the minor allele (A) of rs4149601 is associated with a decreased weight, waist and BMI. Further studies are needed to investigate the relation between *NEDD4L* genetic variants and human body composition traits and muscle strength. Yet, the strong association with the waist that we found makes a predominant effect on lean mass unlikely, since obesity is associated more strongly with excess adipose tissue than with excess muscle mass [20].

The G→A variant of rs4149601 is a common variant of *NEDD4L*, which encodes a new protein (novel C2-domain) that lacks a full length C2-domain [21,22]. It has been speculated that *NEDD4L*, lacking the functionally crucial C2-domain, downregulates ENaC more potently than the protein variants with the intact C2-domain [23]. Consequently, many researchers estimated that the A allele would decrease BP by downregulating Na^+^ re-absorption [6,24]. However, unlike NEDD4, one functional study showed that *NEDD4L*, both with and without the C2-domain, can strongly reduce ENaC activity [25]. Furthermore, in this case, ubiquitination of the epithelial sodium channel could be reduced, due to the novel C2-domain, and the activity of the epithelial sodium channel-Na transport and blood pressure are expected to be increased. Correspondingly, controversial results had been observed in the relationship between SNP rs4149601 and essential hypertension [3,24]. Notably, recent functional studies identified a great extension to the knowledge surrounding *NEDD4L*. First, in addition to targeting sodium channels, *NEDD4L* has also been shown to negatively regulate TGF-β signaling by targeting Smad2 and Smad3 for degradation [26]. Second, dysregulation of TGF-β signaling has been implicated in diseases, such as type 2 diabetes, obesity and cancer [27]. Our results may thus build on a small, but growing body of literature suggesting that the functional variant of rs4149601 in *NEDD4L* may also participate in the regulation of metabolism and be associated with the development of obesity and related phenotypes.

Obesity has become a worldwide epidemic and represents a major risk factor for type 2 diabetes, hypertension, cardiovascular disease and stroke. Few, if any, effective options for treatment and prevention are available. The findings of our study are in line with the initial hypothesis that modulators of *NEDD4L* activity may be associated with obesity and related phenotypes and may thus provide a valuable new strategy for treatment and prevention of obesity and its related diseases. Another attractive aspect of our findings is that the SNP of rs4149601 is a functional variant within the *NEDD4L* gene. However, we have little information on the functional effects of the genetic variants of *NEDD4L* on obesity and related phenotypes; we cannot conclude from our data whether the lower weight, waist and BMI that we observed in carriers of the minor allele of the rs4149601 is associated with increased or decreased *NEDD4L* protein activity. Thus, further studies into underlying mechanisms and body composition are also needed, as well as prospective studies on the relation between *NEDD4L* variants and obesity or related phenotypes.

Beyond these findings, our study also has certain limitations. First of all, analyses were performed in a moderately sized sample, which is underpowered to detect moderate or small effects, underlining the necessity to conduct larger studies. In the present study, for the sample of 256 cases and 636 controls, the power estimates were larger than 90% to detect a log-additive genotype relative risk of 0.65. For the quantitative analyses in the sample of 892 subjects, the power estimate was larger than 85% to detect an additive effect of 0.24 in units of SD of a standard normal distribution (standardized effect size). Thus, all samples were well powered to detect strong effect sizes of disease predisposing variants; moderate or smaller effects might have been missed. However, additional analyses with larger samples are necessary. Indeed, while this manuscript was in preparation, as part of the Genetic Research in Isolated Han Chinese Populations Program in Southern China, similar studies are being carried out in another Han Chinese population. We expect that the findings from the present study will be replicated.

Second, note that our control and case group are both older than 50 years, which might be able to reduce the chances of misclassification errors as opposed to the use of children and adolescents, because younger men might become overweight or obese later on in life. However, it is reasoned that older individuals have longer exposure to putative environmental and genetic influences. Thus, further studies are needed to replicate our findings among young subjects. Third, plasma leptin [28] and insulin [29] levels are important obesity-related phenotypes, which may contribute to the understanding of the physiopathology of the associations among genetic variants and obesity. These data will be collected in the next phase of our study. Finally, in this study, we focused on the relationship between three common variants and obesity or related phenotypes. It is necessary to clarify whether other variants of *NEDD4L* have any effects on obesity and related phenotypes in the future.

## 3. Experimental Section

### 3.1. Ethics Statement

The studies were approved by the Ethical Committees of both the Canton Center for Disease Control and Prevention (Canton CDC) and South Medical University and adhered to the principles of the Declaration of Helsinki. Written informed consent was obtained from each individual enrolled before entry into the study, and all of the procedures were in accordance with institutional guidelines.

### 3.2. Subjects

All of the DNA samples and clinical data for participants in this study were collected from the Genetic Research in Isolated Han Chinese Populations Program in Southern China. The aim of this program was to identify genetic risk factors in the development of chronic disabling disease. For this program, the participants were restricted to local permanent residents of Han Chinese with no mixed marriages within the past three generations, ensuring a completely homogeneous ethnic background. In the present study, we focused on 892 participants, randomly recruited from screening during the ten-month period from March to December 2011 in seven districts of Canton, for whom complete phenotypic, genotypic and genealogical information was available.

The case group was comprised of 256 overweight and obese subjects (mean age ± SD: 53.05 ± 7.83 years; 156 females (60.1%); mean BMI: 27.85 ± 2.66 kg/m^2^). The control group was comprised of 636 healthy lean individuals (mean age ± SD: 56.52 ± 8.18 years; 378 females (59.4%); mean BMI: 21.10 ± 2.65 kg/m^2^). Moreover, our controls reported to have never been overweight or obese earlier in their lives.

### 3.3. Measurements

Weight and height were measured by the same two investigators. Height was measured to the nearest 0.5 cm on a standardized height board. Weight was determined in light underwear and no shoes to the nearest 0.1 kg on a standard physician’s beam scale. BMI was computed as weight in kilograms divided by height in meters squared (kg/m^2^). Overweight and obesity were defined according to the criteria of WHO: normal weight, 18.5 kg/m^2^ ≤ BMI < 25 kg/m^2^; overweight, 25 kg/m^2^ ≤ BMI ≤ 30 kg/m^2^); and obesity, BMI >30 kg/m^2^. Waist circumference was measured to the nearest centimeter with a flexible steel tape measure while the subjects were in a standing position at the end of gentle expiration. All of the participants were asked to avoid alcohol, smoking, coffee, tea and exercise for 30 min before the BP measurements. A standardized mercury sphygmomanometer and appropriate cuff size (regular adult, large or thigh) were used by trained nurses to measure the BP in the subjects’ right arm. The sitting BP was measured 3 times, 30 s apart after 5 min of rest, and the average was defined as the BP level. The average blood pressure of the last two measurements ≥140/90 mmHg or a self-report diagnosis of hypertension was defined as hypertensive.

Blood specimens were drawn after overnight fasting and analyzed for triglycerides (TG), total cholesterol (TC), low-density lipoprotein cholesterol (LDL-c), high-density lipoprotein cholesterol (HDL-c) and glucose. The intra- and inter-assay coefficient variations of these variables were all less than 5%.

### 3.4. SNP Selection and Genotyping

The inclusion criteria of candidate SNPs selection are SNPs among the *NEDD4L* gene, and previous literature has reported the association with essential hypertension and its related phenotypes; SNPs data from the Han Chinese Population included in the HapMap were also used as referred to. Furthermore, SNPs that failed in the assay design were excluded. A total of 3 frequency-validated SNPs were selected for genotyping.

The experimental procedures mentioned in this article have been previously described [30–32]. Briefly, three selected common variants (rs4149601, rs3865418 and rs2288774) were genotyped using the Sequenom MassARRAY matrix-assisted laser desorption ionization-time of flight mass spectrometry platform (Sequenom, San Diego, CA, USA) on genomic DNA isolated from peripheral leukocytes. The first step was to amplify the genomic sequence containing the SNP by a standard PCR protocol, which would produce amplicons 80–120 bp in length. Subsequently, the single-base extension (SBE) reaction was performed on the genomic amplification product using iPlex enzyme and mass-modified terminators. The products of the iPlex reaction were desalted and transferred onto a SpectroCHIP by the MassARRAY nanodispenser. The SpectroCHIP was then analyzed by the MassARRAY Analyzer Compact. All primers used are available on request. For the validity of genotypes, 78 individuals were genotyped in duplicate; concordance was 100%.

### 3.5. Statistical Analyses

Power calculations were performed using the software QUANTO Version 1.2.4 [33] (University of Southern California, Los Angeles, CA, USA; http://hydra.usc.edu/gxe) for all common variants, using an estimated minor allele frequency (MAF) of 0.2 and α = 0.05 (two-sided). A Hardy-Weinberg Equilibrium test was done for each SNP by Pearson’s goodness-of-fit Chi-square test before further analysis, and a *p*-value <0.05 was considered to show significant deviation of the observed genotypes from the Hardy-Weinberg proportions. Linkage disequilibrium (LD) between SNPs was assessed by *D*’ and *r*^2^ values using Haploview version 4.2 [34]. A value of 0 for *D*’ indicates that the examined loci are in fact independent of one another, while a value of 1 demonstrates complete dependency [35].

The odds ratio of being overweight and obese compared with normal weight was assessed using logistic regression. Obesity-related phenotypes of interest were examined for normality and log-transformed in the case of blood parameters (TG, TC, HDL-C, LDL-C and glucose) to achieve an approximately normal distribution. Analyses of all quantitative variables (waist circumference, weight, height, BMI, blood pressure and the log-transformed blood parameters) were performed using a linear regression with SNPStats [36]. The covariates, age, sex, smoking (graded current/former/never), hypertensive status, presence of cardiovascular medications, alcohol consumption (in units per week) and exercise habit (graded non-regular/1- or 2-times per week/three- or more times per week) were considered. Unless otherwise stated, all analyses were performed using a additive genetic model, and all reported *p*-values are nominal, two-sided and not adjusted for multiple testing. Correction for multiple testing was done by Bonferroni’s inequality method wherever applicable and was defined as the *p-*value (single tests) × number of tests. Bonferroni correction was not done for multiple traits, due to the lack of any significant association. Statistical significance was established at a two-tailed value of *p* < 0.05.

## 4. Conclusions

In conclusion, we analyzed three common variants in *NEDD4L*, namely rs2288774, rs3865418 and rs4149601, for association with overweight/obesity and 11 related phenotypes. We found that carriers of the minor allele (A) of rs4149601 have lower weight, waist and BMI and a decreased risk of being overweight/obese. Together with results from the recent studies, these consistent findings warrant research into a potential role for *NEDD4L* modulators in the prevention and treatment of human obesity.

## Supplementary Information



## Figures and Tables

**Table 1:** Interethnic comparisons of allele frequencies for three common single-nucleotide polymorphisms (SNPs) of *NEDD4L* in our subjects with HapMap data.

dbSNP ID	Allele	Present study	HCB a,b	Japanese b	Caucasians b	Blacks b
Rs4149601	G	1369 (79.1%)	80.0%	87.5%	63.3%	63.3%
	A	361 (20.9%)	20.0%	12.5%	36.7%	36.7%
Rs3865418	C	1129 (34.7%)	64.4%	76.7%	55.0%	32.5%
	T	615 (35.3%)	35.6%	23.3%	45.0%	67.5%
Rs2288774	T	1071 (61.1%)	67.8%	69.3%	48.3%	33.3%
	C	683 (38.9%)	32.2%	30.7%	51.7%	66.7%

a HCB, Han Chinese in Beijing, China;

b data from HapMap Data.

**Table 2:** Difference in allele frequency of three selected *NEDD4L* SNPs between cases and controls.

dbSNP ID	Allele major/minor	MAF a		*P*^b^/*P*^c^

		Control	Case	
Rs2288774	T/C	38.7%	39.6%	0.70/NS d
Rs3865418	C/T	34.3%	37.6%	0.18/NS d
Rs4149601	G/A	23.1%	15.3%	**0.0002/0.0006**

a MAF, minor allele frequency;

b *p*-value calculated by Chi-squared test;

c *p*-value after Bonferroni correction;

d NS, Not significant.

**Table 3:** Association analysis of three common SNPs in *NEDD4L* with overweight/obesity.

dbSNP ID	Genotype	*n* (%)		OR (95%CI) a	OR (95%CI) b	*P*^a^/*P*^b^

		Controls	Cases			
Rs2288774	TT	234 (37.4%)	96 (38.2%)	1.00	1.00	
	TC	300 (47.9%)	111 (44.2%)	0.96 (0.71–1.30)	0.95 (0.70–1.29)	0.81/0.73
	CC	92 (14.7%)	44 (17.5%)			

Rs3865418	CC	266 (42.8%)	99 (39.4%)	1.00	1.00	
	CT	284 (45.7%)	115 (45.8%)	1.15 (0.85–1.55)	1.14 (0.84–1.55)	0.36/0.39
	TT	71 (11.4%)	37 (14.7%)			

Rs4149601	GG	367 (59.6%)	185 (74.3%)	**1.00**	**1.00**	
	GA	213 (34.6%)	52 (20.9%)	**0.51 (0.37**–**0.71)**	**0.52 (0.37**–**0.74)**	**<0.0001/<0.0001**
	AA	36 (5.8%)	12 (4.8%)			

OR, odds ratio; CI, confidence interval;

a results from the logistic regression using an additive genetic model;

b results from the logistic regression after adjustment for age, sex, smoking, hypertensive status, alcohol consumption and exercise habit using an additive genetic model.

**Table 4:** Genotype and obesity-related phenotypes association at rs4149601.

Phenotypes	Genotype	*n* (%)	Mean ± SD	Estimate a (95%CI)	*p*-value
Height, cm	GG	552 (63.8%)	161.04 ± 0.40	−0.50(−1.49 to 0.49)	0.33
	GA	265 (30.6%)	161.24 ± 0.54		
	AA	48 (5.6%)	161.02 ± 1.58		
Weight, kg	GG	552 (63.8%)	56.35 ± 0.50	−**2.60(**−**4.01 to** −**1.19)**	**0.0003**
	GA	265 (30.6%)	54.40 ± 0.68		
	AA	48 (5.6%)	54.19 ± 1.52		
Waist, cm	GG	552 (63.8%)	81.66 ± 0.47	−**2.78(**−**4.29 to** −**1.28)**	**0.0015**
	GA	265 (30.6%)	79.17 ± 0.68		
	AA	48 (5.6%)	78.81 ± 1.61		
BMI, kg/m^2^	GG	552 (63.8%)	23.42 ± 0.19	−**0.97(**−**1.56 to** −**0.38)**	**0.0012**
	GA	265 (30.6%)	22.51 ± 0.24		
	AA	48 (5.6%)	22.48 ± 0.48		
SBP, mmHg	GG	552 (63.8%)	138.20 ± 0.88	−0.84(−3.67 to 1.99)	0.56
	GA	265 (30.6%)	137.20 ± 1.23		
	AA	48 (5.6%)	137.02 ± 2.93		
DBP, mmHg	GG	552 (63.8%)	80.99 ± 0.49	−1.29(−2.80 to 0.23)	0.097
	GA	265 (30.6%)	80.10 ± 0.59		
	AA	48 (5.6%)	77.48 ± 1.59		
TG, mmol/L b	GG	552 (63.8%)	2.53 ± 0.37	−0.22(−1.20 to 0.76)	0.66
	GA	265 (30.6%)	2.27 ± 0.11		
	AA	48 (5.6%)	2.47 ± 0.36		
TC, mmol/L b	GG	552 (63.8%)	5.35 ± 0.05	0.14(−0.05 to 0.32)	0.14
	GA	265 (30.6%)	5.52 ± 0.09		
	AA	48 (5.6%)	5.31 ± 0.23		
LDL-c, mmol/L b	GG	552 (63.8%)	3.12 ± 0.03	0.02(−0.09 to 0.13)	0.75
	GA	265 (30.6%)	3.12 ± 0.05		
	AA	48 (5.6%)	3.23 ± 0.13		
HDL-c, mmol/L b	GG	552 (63.8%)	1.33 ± 0.02	0.04(−0.02 to 0.10)	0.23
	GA	265 (30.6%)	1.38 ± 0.03		
	AA	48 (5.6%)	1.31 ± 0.04		
Glucose, mmol/L	GG	552 (63.8%)	5.75 ± 0.06	0.12(−0.08 to 0.33)	0.24
	GA	265 (30.6%)	5.80 ± 0.09		
	AA	48 (5.6%)	6.26 ± 0.26		

BMI, body mass index; SBP, systolic blood pressure; DBP, diastolic blood pressure; TG, triglycerides; TC, total cholesterol; LDL-c, low-density lipoprotein cholesterol; HDL-c, high-density lipoprotein cholesterol;

a effect of one copy of the minor allele in the additive genetic model as determined by linear regression;

b log 10-transformed with age, sex, smoking, presence of cardiovascular medications, alcohol consumption and exercise habit as covariates.

## References

[1] Li N., Wang H., Yang J., Zhou L., Hong J., Guo Y., Luo W., Chang J. (2009). Genetic variation of *NEDD4L* is associated with essential hypertension in female Kazakh general population: A case-control study. BMC Med. Genet.

[2] Russo C.J., Melista E., Cui J., DeStefano A.L., Bakris G.L., Manolis A.J., Gavras H., Baldwin C.T. (2005). Association of *NEDD4L* ubiquitin ligase with essential hypertension. Hypertension.

[3] Luo F., Wang Y., Wang X., Sun K., Zhou X., Hui R. (2009). A functional variant of *NEDD4L* is associated with hypertension, antihypertensive response, and orthostatic hypotension. Hypertension.

[4] Wen H., Lin R., Jiao Y., Wang F., Wang S., Lu D., Qian J., Jin L., Wang X. (2008). Two polymorphisms in *NEDD4L* gene and essential hypertension in Chinese Hans—A population-based case-control study. Clin. Exp. Hypertens.

[5] Araki N., Umemura M., Miyagi Y., Yabana M., Miki Y., Tamura K., Uchino K., Aoki R., Goshima Y., Umemura S. (2008). Expression, transcription, and possible antagonistic interaction of the human *Nedd4L* gene variant: Implications for essential hypertension. Hypertension.

[6] Dahlberg J., Nilsson L.O., von Wowern F., Melander O. (2007). Polymorphism in *NEDD4L* is associated with increased salt sensitivity, reduced levels of P-renin and increased levels of Nt-proANP. PLoS One.

[7] Butterworth M.B. (2010). Regulation of the epithelial sodium channel (ENaC) by membrane trafficking. Biochim. Biophys. Acta.

[8] Flores S.Y., Debonneville C., Staub O. (2003). The role of Nedd4/Nedd4-like dependant ubiquitylation in epithelial transport processes. Pflugers. Arch.

[9] Rotin D., Schild L. (2008). ENaC and its regulatory proteins as drug targets for blood pressure control. Curr. Drug Targets.

[10] Wang H.M., Li N.F., Yao X.G., Hong J., Luo W.L., Chang J.H. (2010). Association of the rs4149601 polymorphism of *NEDD4L* gene with obesity in Xinjiang Kazakh population. Zhonghua Yi Xue Yi Chuan Xue Za Zhi.

[11] Kotsis V., Stabouli S., Papakatsika S., Rizos Z., Parati G. (2010). Mechanisms of obesity-induced hypertension. Hypertens. Res.

[12] Franco V., Oparil S. (2006). Salt sensitivity, a determinant of blood pressure, cardiovascular disease and survival. J. Am. Coll. Nutr.

[13] Bianchi G., Manunta P. (2004). Adducin, renal intermediate phenotypes, and hypertension. Hypertension.

[14] Hollenberg N.K. (1996). Genes, hypertension, and intermediate phenotypes. Curr. Opin. Cardiol.

[15] Wang H., Hong J., Luo W., Chang J., Yao X., Li N. (2011). Relationship between rs3865418 polymorphism of neural precursor cell expressed developmentally downregulated 4 like gene and obesity in Kazakh general population. Zhongguo Yi Xue Ke Xue Yuan Xue Bao.

[16] Qi L., Heredia J.E., Altarejos J.Y., Screaton R., Goebel N., Niessen S., Macleod I.X., Liew C.W., Kulkarni R.N., Bain J. (2006). *TRB3* links the E3 ubiquitin ligase COP1 to lipid metabolism. Science.

[17] Tarpey P.S., Raymond F.L., O’Meara S., Edkins S., Teague J., Butler A., Dicks E., Stevens C., Tofts C., Avis T. (2007). Mutations in CUL4B, which encodes a ubiquitin E3 ligase subunit, cause an X-linked mental retardation syndrome associated with aggressive outbursts, seizures, relative macrocephaly, central obesity, hypogonadism, pes cavus, and tremor. Am. J. Hum. Genet.

[18] Yang B., Kumar S. (2010). *Nedd4* and *Nedd4–2*: Closely related ubiquitin-protein ligases with distinct physiological functions. Cell. Death Differ.

[19] Molero J.C., Turner N., Thien C.B., Langdon W.Y., James D.E., Cooney G.J. (2006). Genetic ablation of the c-Cbl ubiquitin ligase domain results in increased energy expenditure and improved insulin action. Diabetes.

[20] Zillikens M.C., van Meurs J.B., Rivadeneira F., Amin N., Hofman A., Oostra B.A., Sijbrands E.J., Witteman J.C., Pols H.A., van Duijn C.M. (2009). SIRT1 genetic variation is related to BMI and risk of obesity. Diabetes.

[21] Itani O.A., Campbell J.R., Herrero J., Snyder P.M., Thomas C.P. (2003). Alternate promoters and variable splicing lead to *hNedd4–2* isoforms with a C2 domain and varying number of WW domains. Am. J. Physiol. Renal Physiol.

[22] Dunn D.M., Ishigami T., Pankow J., von Niederhausern A., Alder J., Hunt S.C., Leppert M.F., Lalouel J.M., Weiss R.B. (2002). Common variant of human *NEDD4L* activates a cryptic splice site to form a frameshifted transcript. J. Hum. Genet.

[23] Kamynina E., Tauxe C., Staub O. (2001). Distinct characteristics of two human Nedd4 proteins with respect to epithelial Na^+^ channel regulation. Am. J. Physiol. Renal Physiol.

[24] Fava C., von Wowern F., Berglund G., Carlson J., Hedblad B., Rosberg L., Burri P., Almgren P., Melander O. (2006). 24-h ambulatory blood pressure is linked to chromosome 18q21–22 and genetic variation of *NEDD4L* associates with cross-sectional and longitudinal blood pressure in Swedes. Kidney Int.

[25] Itani O.A., Stokes J.B., Thomas C.P. (2005). *Nedd4–2* isoforms differentially associate with ENaC and regulate its activity. Am. J. Physiol. Renal Physiol.

[26] Kuratomi G., Komuro A., Goto K., Shinozaki M., Miyazawa K., Miyazono K., Imamura T. (2005). *NEDD4–2* (neural precursor cell expressed, developmentally down-regulated 4–2) negatively regulates TGF-beta (transforming growth factor-beta) signalling by inducing ubiquitin-mediated degradation of Smad2 and TGF-beta type I receptor. Biochem. J.

[27] Narasimhan S.D., Yen K., Bansal A., Kwon E.S., Padmanabhan S., Tissenbaum H.A. (2011). PDP-1 links the TGF-beta and IIS pathways to regulate longevity, development, and metabolism. PLoS Genet.

[28] Hubacek J.A., Kuthanova L., Bohuslavova R., Adamkova V., Lanska V., Meitinger T., Pfeufer A. (2010). *INSIG2* promoter variant, obesity markers and lipid parameters—No association in a large Slavonic Caucasian population sample. Folia Biol. (Praha).

[29] Vogel C.I., Boes T., Reinehr T., Roth C.L., Scherag S., Scherag A., Hebebrand J., Hinney A. (2011). Common variants near *MC4R*: Exploring gender effects in overweight and obese children and adolescents participating in a lifestyle intervention. Obes. Facts.

[30] Yang X.X., He X.Q., Li F.X., Wu Y.S., Gao Y., Li M. (2012). Risk-Association of DNA Methyltransferases Polymorphisms with Gastric Cancer in the Southern Chinese Population. Int. J. Mol. Sci.

[31] Li F.X., Yang X.X., He X.Q., Hu N.Y., Wu Y.S., Li M. (2012). Association of 10q23 with colorectal cancer in a Chinese population. Mol. Biol. Rep.

[32] Yang X.X., Sun J.Z., Li F.X., Wu Y.S., Du H.Y., Zhu W., Li X.H., Li M. (2012). Aberrant methylation and downregulation of sall3 in human hepatocellular carcinoma. World J. Gastroenterol.

[33] Gauderman W.J., Morrison J.M. (2009). QUANTO 1.1: A computer program for power and sample size calculations for genetic-epidemiology studies.

[34] Barrett J.C., Fry B., Maller J., Daly M.J. (2005). Haploview: Analysis and visualization of LD and haplotype maps. Bioinformatics.

[35] Lewontin R.C. (1964). The interaction of selection and linkage. I. General considerations; Heterotic models. Genetics.

[36] Sole X., Guino E., Valls J., Iniesta R., Moreno V. (2006). SNPStats: A web tool for the analysis of association studies. Bioinformatics.

