# Supplementary material for: A Functional Variant of NEDD4L Is Associated with Obesity and Related Phenotypes in a Han Population of Southern China
Source: Int J Mol Sci. 2013 Apr 2;14(4):7433–44. doi: 10.3390/ijms14047433 (PMC3645694; doi:10.3390/ijms14047433)
Supplement: Supplementary file 1 [file ijms-14-07433-s001.doc]

Supplementary Information

**Table S1.** Genotype and obesity–related phenotypes association at rs2288774.

| **Phenotypes** | **Genotype** | ***n* (%)** | **mean ± SD** | **Estimate ^a^ (95% CI)** | ***p* value** |
| --- | --- | --- | --- | --- | --- |
| Height, cm | TT | 330 (38.6%) | 161.42 ± 0.52 | −0.56 (−1.54 to 0.43) | 0.27 |
|  | TC | 411 (46.8%) | 161.09 ± 0.45 |  |  |
|  | CC | 136 (15.6%) | 161.04 ± 0.83 |  |  |
| Weight, kg | TT | 330 (38.6%) | 56.16 ± 0.62 | −0.91 (−2.33 to 0.51) | 0.21 |
|  | TC | 411 (46.8%) | 55.23 ± 0.58 |  |  |
|  | CC | 136 (15.6%) | 56.18 ± 1.04 |  |  |
| Waist, cm | TT | 330 (38.6%) | 81.19 ± 0.59 | −0.70 (−2.21 to 0.82) | 0.37 |
|  | TC | 411 (46.8%) | 80.50 ± 0.59 |  |  |
|  | CC | 136 (15.6%) | 80.85 ± 0.95 |  |  |
| BMI, kg/m2 | TT | 330 (38.6%) | 23.28 ± 0.26 | −0.33 (−0.92 to 0.26) | 0.28 |
|  | TC | 411 (46.8%) | 22.88 ± 0.20 |  |  |
|  | CC | 136 (15.6%) | 23.30 ± 0.37 |  |  |
| SBP, mmHg | TT | 330 (38.6%) | 137.78 ± 1.12 | 0.27 (−2.51 to 3.05) | 0.85 |
|  | TC | 411 (46.8%) | 137.53 ± 1.02 |  |  |
|  | CC | 136 (15.6%) | 139.32 ± 1.71 |  |  |
| DBP, mmHg | TT | 330 (38.6%) | 80.59 ± 0.64 | −0.09 (−1.58 to 1.40) | 0.91 |
|  | TC | 411 (46.8%) | 80.61 ± 0.53 |  |  |
|  | CC | 136 (15.6%) | 80.18 ± 0.81 |  |  |
| TG, mmol/L ^b^ | TT | 330 (38.6%) | 2.74 ± 0.61 | −0.46 (−1.42 to 0.50) | 0.35 |
|  | TC | 411 (46.8%) | 2.34 ± 0.11 |  |  |
|  | CC | 136 (15.6%) | 2.06 ± 0.12 |  |  |
| TC, mmol/L^b^ | TT | 330 (38.6%) | 5.33 ± 0.07 | 0.15 (−0.03 to 0.33) | 0.11 |
|  | TC | 411 (46.8%) | 5.46 ± 0.07 |  |  |
|  | CC | 136 (15.6%) | 5.49 ± 0.11 |  |  |
| LDL−c, mol/L ^b^ | TT | 330 (38.6%) | 3.06 ± 0.04 | 0.09 (−0.02 to 0.20) | 0.098 |
|  | TC | 411 (46.8%) | 3.14 ± 0.04 |  |  |
|  | CC | 136 (15.6%) | 3.19 ± 0.07 |  |  |
| HDL−c, mol/L ^b^ | TT | 330 (38.6%) | 1.34 ± 0.02 | 0.01 (−0.04 to 0.07) | 0.63 |
|  | TC | 411 (46.8%) | 1.32 ± 0.02 |  |  |
|  | CC | 136 (15.6%) | 1.42 ± 0.05 |  |  |
| Glucose, mol/L | TT | 330 (38.6%) | 5.70 ± 0.08 | 0.14 (−0.06 to 0.34) | 0.18 |
|  | TC | 411 (46.8%) | 5.86 ± 0.07 |  |  |
|  | CC | 136 (15.6%) | 5.77 ± 0.13 |  |  |

BMI, Body mass index; SBP, systolic blood pressure; DBP, Diastolic blood pressure; TG, Triglycerides;
TC, Total cholesterol; LDL−c, Low−density lipoprotein cholesterol; HDL−c, High−density lipoprotein cholesterol; ^a^ Effect of one copy of the minor allele in the additive genetic model as determined by linear regression; ^b^ log 10−transformed with age, sex, smoking, presence of cardiovascular medications, alcohol consumption and exercise habit as covariates.

**Table S2.** Genotype and obesity–related phenotypes association at rs3865418.

| **Phenotypes** | **Genotype** | ***n* (%)** | **mean ± SD** | **Estimate ^a^ (95%CI)** | ***p* value** |
| --- | --- | --- | --- | --- | --- |
| Height, cm | CC | 365 (41.8%) | 161.12 ± 0.51 | 0.20 (−0.78 to 1.17) | 0.69 |
|  | CT | 399 (45.8%) | 161.39 ± 0.46 |  |  |
|  | TT | 108 (12.4%) | 161.23 ± 0.84 |  |  |
| Weight, kg | CC | 365 (41.8%) | 55.21 ± 0.61 | 0.91 (−0.49 to 2.31) | 0.20 |
|  | CT | 399 (45.8%) | 56.26 ± 0.59 |  |  |
|  | TT | 108 (12.4%) | 56.09 ± 1.08 |  |  |
| Waist, cm | CC | 365 (41.8%) | 80.22 ± 0.58 | 1.09 (−0.40 to 2.59) | 0.15 |
|  | CT | 399 (45.8%) | 81.30 ± 0.58 |  |  |
|  | TT | 108 (12.4%) | 81.63 ± 1.07 |  |  |
| BMI, kg/m^2^ | CC | 365 (41.8%) | 22.96 ± 0.25 | 0.23 (−0.35 to 0.81) | 0.44 |
|  | CT | 399 (45.8%) | 23.23 ± 0.20 |  |  |
|  | TT | 108 (12.4%) | 23.21 ± 0.38 |  |  |
| SBP, mmHg | CC | 365 (41.8%) | 138.29 ± 1.13 | −0.69 (−3.40 to 2.01) | 0.62 |
|  | CT | 399 (45.8%) | 137.56 ± 0.95 |  |  |
|  | TT | 108 (12.4%) | 137.58 ± 1.82 |  |  |
| DBP, mmHg | CC | 365 (41.8%) | 80.25 ± 0.64 | 0.41 (−1.05 to 1.86) | 0.58 |
|  | CT | 399 (45.8%) | 80.66 ± 0.50 |  |  |
|  | TT | 108 (12.4%) | 80.69 ± 0.81 |  |  |
| TG, mmol/L ^b^ | CC | 365 (41.8%) | 2.68 ± 0.55 | −0.40 (−1.34 to 0.55) | 0.41 |
|  | CT | 399 (45.8%) | 2.27 ± 0.10 |  |  |
|  | TT | 108 (12.4%) | 2.37 ± 0.26 |  |  |
| TC, mmol/L ^b^ | CC | 365 (41.8%) | 5.41 ± 0.07 | 0.02 (−0.16 to 0.20) | 0.86 |
|  | CT | 399 (45.8%) | 5.45 ± 0.07 |  |  |
|  | TT | 108 (12.4%) | 5.36 ± 0.11 |  |  |
| LDL−c, mmol/L ^b^ | CC | 365 (41.8%) | 3.10 ± 0.04 | 0.02 (−0.08 to 0.12) | 0.71 |
|  | CT | 399 (45.8%) | 3.08 ± 0.04 |  |  |
|  | TT | 108 (12.4%) | 3.29 ± 0.08 |  |  |
| HDL−c, mol/L^b^ | CC | 365 (41.8%) | 1.36 ± 0.02 | −0.02 (−0.08 to 0.04) | 0.46 |
|  | CT | 399 (45.8%) | 1.34 ± 0.02 |  |  |
|  | TT | 108 (12.4%) | 1.30 ± 0.03 |  |  |
| Glucose, mol/L | CC | 365 (41.8%) | 5.74 ± 0.07 | 0.08 (−0.12 to 0.28) | 0.42 |
|  | CT | 399 (45.8%) | 5.78 ± 0.07 |  |  |
|  | TT | 108 (12.4%) | 6.00 ± 0.16 |  |  |

BMI, Body mass index; SBP, systolic blood pressure; DBP, Diastolic blood pressure; TG, Triglycerides;
TC, Total cholesterol; LDL−c, Low−density lipoprotein cholesterol; HDL−c, High−density lipoprotein cholesterol; ^a^ Effect of one copy of the minor allele in the additive genetic model as determined by linear regression; ^b^ log 10−transformed with age, sex, smoking, presence of cardiovascular medications, alcohol consumption and exercise habit as covariates.

© 2013 by the authors; licensee MDPI, Basel, Switzerland. This article is an open access article distributed under the terms and conditions of the Creative Commons Attribution license (http://creativecommons.org/licenses/by/3.0/).
